# Crystal Structure of the Human SUV39H1 Chromodomain and Its Recognition of Histone H3K9me2/3

**DOI:** 10.1371/journal.pone.0052977

**Published:** 2012-12-28

**Authors:** Tao Wang, Chao Xu, Yanli Liu, Kai Fan, Zhihong Li, Xing Sun, Hui Ouyang, Xuecheng Zhang, Jiahai Zhang, Yanjun Li, Farrell MacKenzie, Jinrong Min, Xiaoming Tu

**Affiliations:** 1 Hefei National Laboratory for Physical Sciences at Microscale, School of Life Science, University of Science and Technology of China, Hefei, Anhui, People’s Republic of China; 2 Structural Genomics Consortium and Department of Physiology, University of Toronto, Toronto, Ontario, Canada; 3 Hubei Key Laboratory of Genetic Regulation and Integrative Biology, College of Life Science, Huazhong Normal University, Wuhan, People's Republic of China; 4 School of Life Sciences, Anhui University, Hefei, Anhui, People’s Republic of China; Bellvitge Biomedical Research Institute (IDIBELL), Spain

## Abstract

SUV39H1, the first identified histone lysine methyltransferase in human, is involved in chromatin modification and gene regulation. SUV39H1 contains a chromodomain in its N-terminus, which potentially plays a role in methyl-lysine recognition and SUV39H1 targeting. In this study, the structure of the chromodomain of human SUV39H1 was determined by X-ray crystallography. The SUV39H1 chromodomain displays a generally conserved structure fold compared with other solved chromodomains. However, different from other chromodomains, the SUV39H1 chromodomain possesses a much longer helix at its C-terminus. Furthermore, the SUV39H1 chromodomain was shown to recognize histone H3K9me2/3 specifically.

## Introduction

In eukaryote, histone modifications play an important role in regulating gene expression in the native chromatin context. The amino-terminal tails of nucleosomal histones, protruding away from the nucleosome core, are amenable to several forms of posttranslational modifications such as methylation, acetylation, phosphorylation, ADP ribosylation, and ubiquitination [1]. As an abundant epigenetic modification, histone lysine methylation is essential for the organization and function of chromatin. The methylation patterns have been associated with distinct chromatin states and are proposed to be the major epigenetic marks that could extend the genetic code by regulating the chromatin structure in a heritable manner [2].

The human SUV39H1, a histone H3K9 methyltransfearse, is the first histone lysine methyltransferase (HMT) identified, which, together with SUV39H2, are the mammalian homologs of Drosophila Su(var)3-9 and *Schizosaccharomyces pombe* Clr4. A direct consequence of this modification is the creation of a high-affinity binding site for heterochromatin protein 1 (HP1), which together with other proteins induces chromatin packaging and gene silencing [3], [4]. In addition to initiating the formation of large heterochromatin regions, SUV39H1 is also involved in repressing the transcription of specific genes. It interacts with DNA-binding proteins involved in the leukemogenesis such as AML1 and PML-RARα, promoting the silencing of their target genes [5], [6].

SUV39H1 possesses a SET domain at its C-terminus, which performs the catalytic activity, and a chromodomain at its N-terminus. The chromodomain is a conserved motif containing about 50 amino acids and is identified as a module to target proteins to specific chromosomal loci [7]. The chromodomain family displays a broad range of activities, including methyl-lysine histone, DNA and RNA binding [8]–[10]. It was revealed that the chromodomain of SUV39H1 is essential for the catalytic activity of SUV39H1 [11]. Mutation and deletion of the chromodomain of SUV39H1 impaired its enzyme activity in spite of the presence of an intact catalytic SET domain [11]. Presumably binding methylated histones by the chromodomain of SUV39H1 plays a critical role in targeting the catalytic activity of SUV39H1.

Here we report the crystal structure of the chromodomain of human SUV39H1. The structure exhibits a fold similar to other solved chromodomain structures. Furthermore, we identify that the SUV39H1 chromodomain is able to recognize H3K9me2/3 specifically by fluorescence polarization binding assay.

## Results

### The SUV39H1 Chromodomain Exhibits Moderate Sequence Similarity and Conserved Structure Compared with other Chromodomains

**Figure 1:**
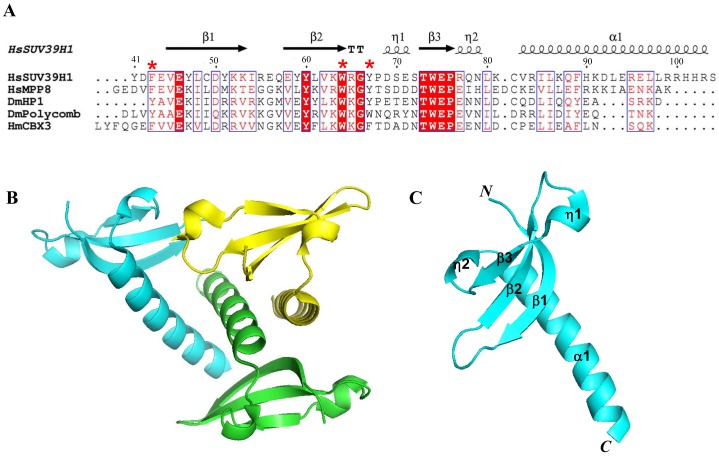
Sequence alignment of chromodomains and the crystal structure of human SUV39H1 chromodomain. A: Alignment of chromodomain sequences of human SUV39H1, human MPP8, *Drosophila* HP1, *Drosophila* polycomb protein and human chromobox homologs CBX3. HsSUV39H1, human SUV39H1 chromodomain; HsMPP8, human MPP8 chromodomain; DmHP1, *Drosophila melanogaster* HP1 chromodomain; DmPolycomb, *Drosophila melanogaster* polycomb protein chromodomain; HsCBX3, human chromobox homolog 3, HP1γ. Secondary structures of the SUV39H1 chromodomain are labeled at the top and the aromatic cage residues are marked by as well. B: The crystallographic asymmetric unit of human SUV39H1 chromodomain, colored in cyan, green and yellow, respectively. C: The ribbon diagram of human SUV39H1 chromodomain monomer colored in cyan and secondary structures are labeled.

Sequence alignment analysis showed that the human SUV39H1 chromodomain displays moderate sequence identity and similarity in comparison with other chromodomains (Fig. 1A). The SUV39H1 chromodomain shares about 30% sequence identity and about 40% sequence similarity with other chromodomains, including human and *Drosophila* HP1 and Polycomb proteins, which are the founding members of chromodomains and whose structures have been determined [8], [12]–[15]. Nevertheless, the SUV39H1 chromodomain shows highly conserved structure features shared in the chromodomain family, including the aromatic cage residues that implies their potential ability in methyl-lysine histone binding (Fig. 1A).

### Overall Structure of SUV39H1 Chromodomain

The crystal structure of the human SUV39H1 chromodomain (aa 44–106) was determined at a resolution of 2.2 Å, and deposited in the Protein Data Bank with an accession number 3MTS. The human SUV39H1 chromodomain, which contains 3 independent molecules in an asymmetric unit, adopts a canonical chromodomain architecture (Fig. 1B/C). However, it exists as a monomer in solution. The fold is composed of an N-terminal SH3-like β-barrel, followed by a long C-terminal helix α1(residues 82–100). The β-barrel consists of a sheet with three antiparalled strands β1 (residues 45–53), β2 (residues 58–64) and β3 (residues 73–76).

### Structural Comparison between Human SUV39H1 Chromodomain and other Chromodomain Family Members

**Figure 2:**
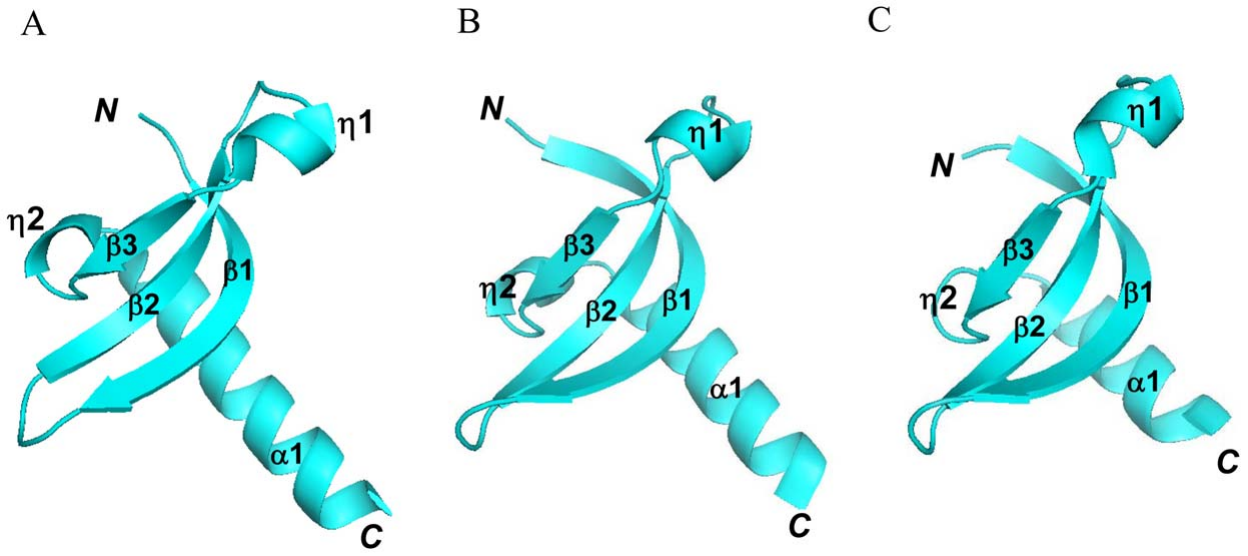
Structural comparison between human SUV39H1 chromodomain and other chromodomains, which are all colored in cyan A: human SUV39H1 chromodomain (PDB ID: 3MTS). B: human MPP8 chromodomain (PDB ID: 3R93) C: *Drosophila* HP1 chromodomain (PDB ID: 1KNE).

Several structural homologs of the SUV39H1 chromodomain were identified by DALI21 (http://ekhidna.biocenter.helsinki.fi/dali_server). The best matched structure identified in the PDB library is that of the chromodomain of MPP8 (PDB code 3R93) [14], with Z = 9.6, RMSD = 1.2 Å, and a sequence identity of 31%. The Z-score and RMSD between the SUV39H1 chromodomain and the *Drosophila* HP1 chromodomain (PDB code 1KNE) are 9.4 and 1.0 Å, respectively, which demonstrates structure conservation between the two structures. Therefore, except for the longer helix α1, the human SUV39H1 chromodomain (Fig. 2A) is structurally very similar to other determined chromodomains (Fig. 2B/C). Interestingly, the chromodomain of the human SUV39H1 we crystallized lacks the first aromatic residue F43 of the aromatic cage. Thus the two aromatic residues, W64 and Y67, in the loop between β2 and β3 form a partial aromatic cage, which is a conserved structural feature among chromodomain proteins and other Royal family members [16]. This may implicate that the cage structural element is not essential for fold stability. The aromatic cage has been widely utilized for recognizing methylated lysine or arginine of proteins, such as the tudor domain of SGF29, which binds histone H3K4me2/3 and targets the SAGA complex [17], the tudor domain of SND1, which binds arginine methylated PIWI proteins and recruits its associated RNA cleavage activity [18], the MBT domain of L3MBTL1/2, which recognizes lower methyated lysine histones [19], [20], the chromo barrel domain of Eaf3, which is a subunit of the NuA4 histone acetyltransferase complex and recognizes methylated H3K36 [21] and the WD40 domain of EED, which is a histone H3K27 reader and a compoent of PRC2 complex [22], [23]. In the next section, we are going to discuss more regarding the histone binding ability of the partial chromodomain of SUV39H1.

Another conserved structural feature of the chromdomain family is that it contains a hydrophobic core consisting of the residues V45, L48, Y60, V62, W64, L80, I85 and L86. All of these residues are also conserved in the chromodomain family (Fig. 1A). Interestingly, the potential peptide binding groove formed by the SUV39H1 chromdomain β sheet is reminiscent of the binding grove identified in the PTB and PDZ domains [24]. All these observations revealed that the human SUV39H1 chromodomain adopts an overall structure similar to that of the other chromodomain family members and contains conserved residues, which form a compact core with a aromatic cage crucial for binding methyllysine [16].

### SUV39H1 Chromodomain Recognizes H3K9me3 by Fluorescence Polarization Assays

**Figure 3:**
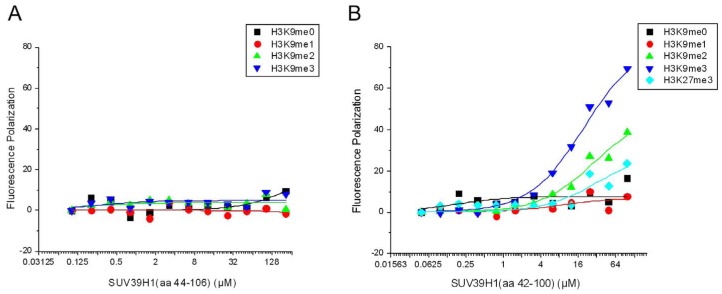
Kinetic analysis of interactions between SUV39H1 chromodomain and H3K9me3/2/1/0 or H3K27me3 peptide was performed by fluorescence polarization assay. Diagrams of different H3 peptides are indicated by different symbols. A: Kinetic analysis of interactions between truncated SUV39H1 chromodomain (aa 44–106) and H3K9me3/2/1/0 peptide. B: Kinetic analysis of interactions between complete SUV39H1 chromodomain (aa 42–100) and H3K9me3/2/1/0 or H3K27me3 peptide.

Because the chromodomain we crystallized contains an incomplete aromatic cage, we next examine if the deletion of the first aromatic residue F43 in SUV39H1 affects its methyl-lysine histone binding. We purified two SUV39H1 constructs including the one used for crystallization (aa 44–106) and the one containing a complete aromatic cage (aa 42–100). By means of fluorescence polarization assay, we found that the partial chromodomain does not show detectable binding to any histone H3K9 peptides (Fig. 3A, Table 1). The importance of a complete aromatic cage has been show previously for CDYL, which lacks the first aromatic residue and lacks histone binding as well [25]. Not surprisingly, the complete chromodomain of SUV39H1 specifically binds to histone H3K9me2/3 with a K_d_ of 20±4 µM for H3K9me3, and a K_d_ of 29±12 µM for H3K9me2 (Fig. 3B, Table1). It does not show detectable binding to histone H3K9me0/1 (Fig. 3B, Table 1). In addition, the SUV39H1 chromodomain just shows very weak binding to histone H3K27me3 (Fig. 3B, Table 1). Taken together, the SUV39H1 chromodomain specifically recognizes histone H3K9me2/3.

**Table 1:** Binding constants of human SUV39H1 chromodomains and H3 histone peptides determined by fluorescence polarization.

Peptides	K_d_ (µM) for SUV39H1 (42–100)	K_d_ (µM) for SUV39H1 (44–106)
H31–15K9me3	20±4	*NB
H31–15K9me2	29±12	*NB
H31–15K9me1	*NB	*NB
H31–15K9me0	*NB	*NB

* NB: No detectable binding from FP experiments.

## Discussion

SUV39H1 is the first identified histone lysine methyltransferase (HMT) in human [26], [27]. It catalyzes di- and tri-methylation of lysine 9 of histone H3, which are related to chromatin packaging and gene silencing [3], [4]. SUV39H1 possesses a chromodomain at its N-terminus and a SET domain at its C-terminus. Although the SET domain performs the catalytic activity, the chromodomain of SUV39H1 is vital for the catalytic activity of SUV39H1 [11].

In this study we determined the 3D structure of the chromodomain of human SUV39H1. It exhibits a three-dimensional fold similar to that of the determined structures of other chromodomains. However, it possesses a much longer helix at its C-terminus, which is different from other chromodomains. Additionally, we showed that the SUV39H1 chromodomain specifically binds to H3K9me2/3. The chromodomains of HP1 and MPP8 preferentially bind to H3K9me3 [14]. We also confirmed that the SUV39H1 chromodomain also binds H3K9me3 and H3K9me2, albeit the latter binds about 1.5 fold weaker. However, no strong interactions between the chromodomain of human SUV39H1 and H3K27me3 were observed, which indicates that the chromodomain of human SUV39H1 would bind H3K9me2/3 specifically. Residues Q5-S10 (QTARKS) in the H3K9 peptide are critical for the interactions between the chromodomains and the H3K9 peptide. H3K9 and H3K27 share the sequence of ARKS, however, the sequence in H3K27 is KA instead of QT preceding the conserved ARKS motif. This difference was identified to be important for distinguishing H3K9 from H3K27 [14]. Therefore, our binding results show that the SUV39H1 chromodomain specifically recognizes the histone H3K9me2/3 mark, and the detailed molecular mechanism of how the SUV39H1 chromodomain specifically recognizes histone H3K9me2/3 warrant future complex structure determination.

**Figure 4:**
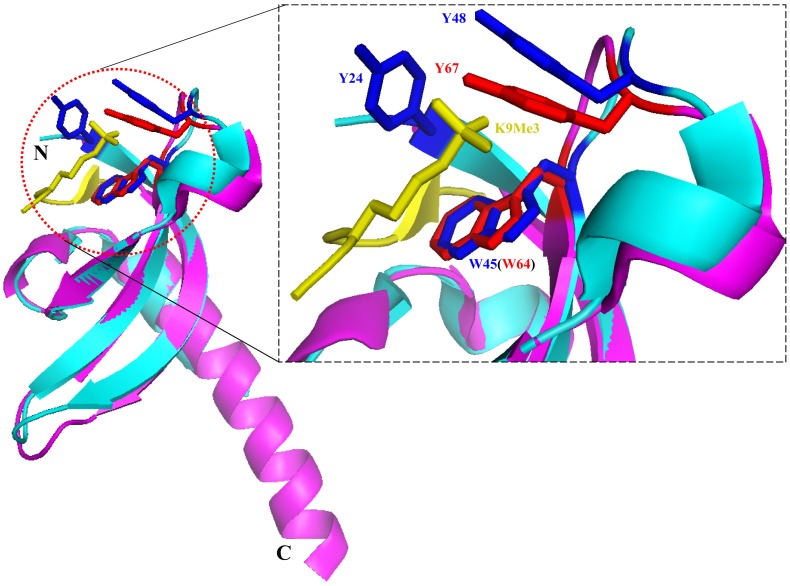
Hypothetical model of H3K9me3 binding by human SUV39H1 chromodomain. The structures of human SUV39H1 and *Drosophila melanogaster* HP1 (PDB: 1KNE) chromodomains are aligned and shown in magenta and cyan, respectively. Y24, W45 and Y48 of *Drosophila melanogaster* HP1 chromodomain that are critical for H3K9me3 binding are shown as sticks in blue. The corresponding residues, W64 and Y67 of human SUV39H1 chromodomain, are shown as sticks in red. H3K9me3 peptide is shown in yellow with trimethylated lysine 9 shown as sticks.

It has been reported that the chromodomain of human SUV39H1 is essential for the protein’s catalytic activity. Deletion of the chromodomain or point mutation of the conserved amino acids, W64A or Y67A, of the chromodomain in SUV39H1 impaired the enzyme activity even if the catalytic SET domain is intact [11]. Consistently, it has been show that the chromodomain of Clr4, which is a *Schizosaccharomyces pombe* homolog of human SUV39H1, binds specifically to H3K9me and is responsible for its H3K9 methylation activity targeting and the spreading of heterochromatin [11], [28]. It is tempting to speculate that the chromodomain of SUV39H1 would play a similar role to that of the Clr4 chromodomain. Sequence alignment and structural comparison demonstrated that the human SUV39H1 chromodomain is similar to the *Drosophila* HP1 chromodomain, which was the first chromodomain characterized structurally at an atomic level and provided insight into its function as a methylated histone lysine binding domain [15], [29]. The methylated ligand is coordinated by three aromatic residues that form an open “cage”, which is partially hydrophobic around the moiety. The sequence alignment revealed that there are approximately 29% sequence identities and 42% similarity between the human SUV39H1 chromodomain and *Drosophila* HP1 chromodomains (Fig. 1A). Additionally, their 3D structures are also similar to each other. Especially, the key residues responsible for the recognition of H3K9me3, W45 and Y48 in *Drosophila* HP1 chromodomain, are conserved in the two sequences. The residues are located in the similar region of the structures and the side chains protrude from the backbone in a similar orientation (Fig. 1A & Fig. 4). Therefore, the corresponding residues in human SUV39H1 chromodomain, W64 and Y67, are supposed to be also important for methylated histone lysine binding. Sequence and structure similarity between the two chromodomains, and especially the conservation of the key residues involved in the “cage”, together suggest that they may interact with histone H3K9me3 in a similar way.

## Materials and Methods

### Protein Expression and Purification

The human SUV39H1 chromodomain (residues 44–106 or 42–100) was subcloned into pET28a-MHL vector. SUV39H1 chromodomain for crystallization and binding assays are the fragments from residue 44 to 106 and from residue 42 to 100, respectively. The recombinant protein was over-expressed at 18°C as an N-terminal His6-tagged protein in E. coli BL21 (DE3) Codon plus RIL (Stratagene) and was purified by HiTrap Ni column [30]. The obtained proteins were further purified on a HiLoad 16/60 Superdex 200 prep-grade column. The final sample for crystallization contained 10 mg/ml SUV39H1 chromodomain and 3.5 M Na Formate, 0.1 M Bis-Tris Propane (pH 7.0) for crystallization.

### Data Collection, Structure Determination and Refinement

Diffraction data were collected at 100 K on beamline 23-ID-B (GM/CA-CAT, Advanced Photon Source, Argonne National Laboratory) using a MARMOSAIC 300 CCD detector. The crystal belonged to space group R32, with unit cell parameters a = b = 99.5, c = 118.3 Å, and diffracted to 2.2 Å resolution. The data were integrated and scaled using the HKL2000 software package [31]. The structure was solved by molecular replacement using the crystal structure of the chromo domain of HP1 from Drosophila melanogaster (PDB 1KNE) as a search model and the program Phaser as implemented in the Phenix program suite [32], [33]. Following several alternate cycles of restrained refinement and manual rebuilding using COOT, the improved model revealed clear electron densities allowing placement of ordered solvent molecules [34]. All refinement steps were performed using REFMAC in the CCP4 program suite [35], [36]. During the final cycles of model building, TLS parameterization was included in the refinement which comprised three protein chains and solvent molecules [37], [38]. Data collection and refinement statistics are summarized in Table 2. The stereochemical quality of the final model was validated by PROCHECK online (http://nihserver. mbi.ucla.edu/SAVES_3/).

**Table 2:** Data collection and refinement statistics.

Data collection	
Space group	*R*32
Cell dimensions	
a, b, c (Å)	99.5, 99.5, 118.3
α, β, γ (°)	90, 90, 120
Wavelength (Å)	0.97944
Resolution (Å)	50.00–2.20 (2.28–2.20)
R_merge_ (%)	8.4 (50.2)
*I/σI*	25.6 (4.6)
Completeness (%)	100.0 (100.0)
Redundancy	9.3 (9.3)
**Refinement**	
Resolution (Å)	34.8–2.2
No. reflections	11,102
*R*_work_/*R*_free_	20.8/24.4
No. atoms	
Protein	1565
Water	32
Average B-factors (Å^2^)	
Protein	25.1
Water	17.8
R.m.s. deviations	
Bond lengths (Å)	0.012
Bond angles (°)	1.290
**Ramachandran plot, % of all residues**	
Most favored regions	94.6
Additional allowed regions	4.2
Generously allowed regions	0.6
Disallowed regions	0.6

Values in parentheses are for the highest-resolution shell.

### Fluorescence Polarization

All peptides used for fluorescence polarization measurements were synthesized by Tufts University Core Services. The assay was performed in 10 µL at a constant fluorescence labeled-peptide concentration of 40 nM and increasing amounts of SUV39H1 (residues 42–100 or 44–106) at concentrations ranging from low to high micromolar in a buffer of 20 mM Tris • HCl, pH 8.5 or 7.5, 150 mM NaCl, 1 mM DTT, and 0.01%Tween-X-100. The assay was performed in 384-well plates, using a Synergy 2 microplate reader (BioTek). An excitation wave length of 485 nm and an emission wave length of 528 nm were used. The data were corrected for background of the free-labeled peptides. To determine the K_d_ values, the data were fit to a hyperbolic function using Sigma Plot software (Systat Software, Inc.).
